# Treatment Combinations with DNA Vaccines for the Treatment of Metastatic Castration-Resistant Prostate Cancer (mCRPC)

**DOI:** 10.3390/cancers12102831

**Published:** 2020-09-30

**Authors:** Melissa Gamat-Huber, Donghwan Jeon, Laura E. Johnson, Jena E. Moseman, Anusha Muralidhar, Hemanth K. Potluri, Ichwaku Rastogi, Ellen Wargowski, Christopher D. Zahm, Douglas G. McNeel

**Affiliations:** University of Wisconsin Carbone Cancer Center, University of Wisconsin-Madison, Madison, WI 53726, USA; gamat@wisc.edu (M.G.-H.); donghwan.jeon@wisc.edu (D.J.); lej@medicine.wisc.edu (L.E.J.); jemoseman@wisc.edu (J.E.M.); anusha.muralidhar@wisc.edu (A.M.); hpotluri@wisc.edu (H.K.P.); irastogi@wisc.edu (I.R.); ewargows@medicine.wisc.edu (E.W.); chris.zahm@wisc.edu (C.D.Z.)

**Keywords:** DNA vaccine, combination therapy, prostate cancer, androgen deprivation, chemotherapy, radiation, immune checkpoint blockade, TLR agonist, IDO inhibitor

## Abstract

**Simple Summary:**

The only vaccine approved by FDA as a treatment for cancer is sipuleucel-T, a therapy for patients with metastatic castration-resistant prostate cancer (mCRPC). Most investigators studying anti-tumor vaccines believe they will be most effective as parts of combination therapies, rather than used alone. Unfortunately, the cost and complexity of sipuleucel-T makes it difficult to feasibly be used in combination with many other agents. In this review article we discuss the use of DNA vaccines as a simpler vaccine approach that has demonstrated efficacy in several animal species. We discuss the use of DNA vaccines in combination with traditional treatments for mCRPC, and other immune-modulating treatments, in preclinical and early clinical trials for patients with mCRPC.

**Abstract:**

Metastatic castration-resistant prostate cancer (mCRPC) is a challenging disease to treat, with poor outcomes for patients. One antitumor vaccine, sipuleucel-T, has been approved as a treatment for mCRPC. DNA vaccines are another form of immunotherapy under investigation. DNA immunizations elicit antigen-specific T cells that cause tumor cell lysis, which should translate to meaningful clinical responses. They are easily amenable to design alterations, scalable for large-scale manufacturing, and thermo-stable for easy transport and distribution. Hence, they offer advantages over other vaccine formulations. However, clinical trials with DNA vaccines as a monotherapy have shown only modest clinical effects against tumors. Standard therapies for CRPC including androgen-targeted therapies, radiation therapy and chemotherapy all have immunomodulatory effects, which combined with immunotherapies such as DNA vaccines, could potentially improve treatment. In addition, many investigational drugs are being developed which can augment antitumor immunity, and together with DNA vaccines can further enhance antitumor responses in preclinical models. We reviewed the literature available prior to July 2020 exploring the use of DNA vaccines in the treatment of prostate cancer. We also examined various approved and experimental therapies that could be combined with DNA vaccines to potentially improve their antitumor efficacy as treatments for mCRPC.

## 1. Introduction

Prostate cancer has been generally viewed as an immunologically “cold” tumor (i.e., devoid of infiltrating lymphocytes), because trials evaluating T-cell checkpoint blockade therapies, including anti-PD-1 or anti-CTLA-4, have shown little benefit for all but a small number of patients with prostate cancer [1,2,3,4]. This lack of response has been partly attributed to lower numbers of tumor-infiltrating CD8+ T cells in prostate tumors relative to many other solid tumor types that benefit from checkpoint blockade therapy [5]. Prostate cancers also have lower mutational burdens, suggesting a lower number of potential tumor-specific mutation-associated neoantigens for CD8+ T cells to recognize [6]. Although the presence of tumor infiltrating CD8+ T cells has been associated with favorable long-term outcomes for most tumor types, this association has been controversial in prostate cancer. In fact, several reports have associated a higher frequency of CD8+ prostate-infiltrating lymphocytes with shorter time to disease progression [7,8]. Emerging research also suggests that the prostate tumor microenvironment contains elevated levels of myeloid-derived suppressor cells (MDSCs) and indoleamine 2,3 dioxygenase (IDO), contributing to an immunosuppressive environment [9,10]. Together, these findings have suggested that the microenvironments of prostate tumors may be different from those of other solid tumors and that additional immune regulatory cells, cytokines, or other metabolic factors such as hypoxia may affect the function of tumor-infiltrating CD8+ T cells and the response of prostate tumors to immune therapies [11].

Despite the poor response of prostate tumors to T-cell checkpoint blockade therapies, prostate cancer clearly can respond to immune-targeted therapies. Vaccines, agents able to activate and expand tumor-associated T cells, have demonstrated clinical activity in prostate cancer [12,13,14,15,16]. In fact, only one therapeutic cancer vaccine has been FDA-approved for human use, sipuleucel-T (Sip-T), which is an autologous cellular cancer vaccine for patients with mCRPC [17]. Sip-T was approved on the basis of randomized clinical trials demonstrating improved overall survival of treated patients [18,19,20]. In contrast, many antitumor vaccines investigated for patients with melanoma, a disease characterized by many more tumor-infiltrating CD8+ lymphocytes, have failed to demonstrate significant clinical benefit to mCRPC patients. This suggests that vaccines may skew the population of CD8+ T cells, or in some cases, permit infiltration of tumors by CD8+ T cells, an approach that might work best in tumors with few tumor-infiltrating lymphocytes. Patients with newly diagnosed prostate cancer who were treated with Sip-T prior to prostatectomy had significantly more tumor-infiltrating T cells [21]. This suggests that agents, such as T-cell checkpoint molecules that can modulate the function of these tumor-infiltrating lymphocytes, might best be combined with antitumor vaccines. This approach was tested in a murine model of prostate cancer, in which TRAMP mice were treated with the cellular vaccine GVAX (a cellular vaccine made from gene-modified tumor cells), anti-CTLA-4 blocking antibody, or the combination. Decreased tumor grade and increased lymphocytic infiltration of tumors was observed only with the combination treatment [22]. This combination was then evaluated in a small clinical trial in which patients with advanced prostate cancer were treated with the GVAX vaccine in combination with ipilimumab [23]. Specifically, 20% of patients experienced 50% or greater declines in serum PSA levels, suggesting clinical activity. Unfortunately, GVAX alone was not further pursued after early failures in two phase 3 trials [24,25]. In one study, patients were treated with GVAX or docetaxel and prednisone, however, it was terminated early due to a data monitoring committee determination that the study had less than 30% chance of meeting its primary endpoint of increased overall survival [24]. In another study, GVAX combined with docetaxel was compared to docetaxel with prednisone in patients with CRPC, where it was determined that overall survival was significantly shorter in the GVAX arm [25]. Due to the decision to not further pursue GVAX as a treatment, the combination approach of GVAX with ipilimumab was not further explored. Notwithstanding, this demonstrates that combination approaches, using antitumor vaccines with agents able to modulate the infiltration or activity of immune effector cells elicited with vaccination, are reasonable for further evaluation in clinical trials.

Although Sip-T has shown a significant but arguably modest effect in the treatment of mCRPC, and could theoretically be combined with other agents, the cost associated with this treatment makes it less accessible to the majority of patients and makes combination approaches potentially prohibitively costly [26]. DNA vaccines are significantly cheaper, can be used off-the-shelf, and several have already been approved for use in veterinary medicine [27]. Many therapies that are currently in use and in development also have immunomodulatory effects, which can be leveraged to further enhance the antitumor efficacy of DNA vaccines. Combining DNA vaccines with other therapies is consequently a valid approach for the treatment of mCRPC, and these combinations will be the focus of this review.

## 2. DNA Vaccines as Treatments for Prostate Cancer

Cancer vaccines act by inducing a specific, and ideally long-lasting, immune response against tumor antigens. Various types of cancer vaccines have been tested, with varied mechanisms to elicit an immune response to either arrest cancer progression or prevent tumor recurrence. In the case of prostate cancer, these have included cell-based vaccines, such as dendritic cell vaccines (e.g., Sip-T) or whole tumor cells (e.g., GVAX), protein/peptide vaccines, viral/bacterial-based vaccines (e.g., PROSTVAC, Bavarian Nordic Immunotherapies), and gene-based vaccines, including RNA and DNA vaccines [28]. Vaccines can target antigens shared by tumors from different individuals, or alternatively, neoantigen vaccines are also being explored, which are specific to mutations arising in individual tumors. Theoretically, mutation-associated neoantigen vaccines should be less susceptible to pre-existing T cell tolerance, because the antigens targeted should not be presented in the thymus [28]. However, the small number of tumor-specific mutations in prostate cancer may ultimately limit the feasibility of neoantigen vaccines as an approach for prostate cancer. Although several vaccine approaches have been evaluated in clinical trials for patients with prostate cancer, and reviewed elsewhere, our focus here will be on DNA vaccines.

DNA vaccines are simple vehicles for in vivo transfection and antigen production, consisting of a circular piece of DNA that encodes the antigen of interest under the control of a eukaryotic promoter [29]. DNA vaccines have several advantages over other vaccine platforms. They are easily amenable to design alterations, scalable for large-scale manufacturing, not infectious, not restricted to individuals of a defined MHC type, and thermostable for easy transport and distribution. DNA vaccines are administered by one of several delivery methods such as gene gun to the epidermis, intramuscular injection [30], or intradermal injection. Conceptually, transfected dendritic cells, or dendritic cells cross-presenting antigen produced by bystander transfected cells, travel from the site of delivery to draining lymph nodes, where antigen presentation and T-cell activation occur [31]. Intramuscular injection results in transfection of myocytes that, lacking costimulatory molecules and MHC II molecules, transfer antigen to professional antigen-presenting cells, which can cross-present the antigen to CD8+ T cells [32]. The route of immunization can affect the resulting type of immune response [33].

DNA vaccines are currently not approved for use in humans but have been approved for the treatment of West Nile virus in horses [34] and canine melanoma [35]. However, human DNA vaccines are an active field of research, with clinical trials ongoing in a variety of cancers and infectious disease [36,37,38,39,40,41,42,43].

Identifying target antigens is critical in vaccine design. As described above, while tumor-specific mutation-associated neoantigens are being explored, most prior evaluation of prostate cancer vaccines has focused on antigens specific to prostate cancer and shared by multiple individuals. Ideally, the target should be only expressed in the prostate (or more specifically, the tumor) or at the least highly expressed in the prostate tumor compared to regular tissues to minimize off-tumor cytotoxicity. At least four proteins have been identified as potential immune targets for DNA vaccines that are currently or have been explored in clinical trials for patients with prostate cancer: prostatic acid phosphatase (PAP), prostate-specific antigen (PSA), prostate-specific membrane antigen (PSMA), and the androgen receptor (AR) [12,15,44,45]. Preclinical studies investigating the use of DNA vaccines for prostate cancer have recently been reviewed by our group and others [27,28,46]. A summary of previous and ongoing clinical trials conducted with DNA vaccines in patients with prostate cancer is shown in Table 1.

The most studied antigen in human prostate cancer vaccine trials is prostatic acid phosphatase (PAP), the same target of the sipuleucel-T vaccine. PAP is expressed at high levels in the epithelium in prostate cancer patients [54]. In a Phase 1/2 dose-escalation study (NCT00582140), patients received pTVG-HP at three different doses: 100, 500, and 1500 μg at 14-day intervals for six doses. The vaccine was well tolerated, and of the 22 patients on study, 6 patients developed at least a threefold increase in PAP-specific CD4+ proliferative T cells, and 3 patients developed at least a threefold increase in PAP-specific CD8+ proliferative T cells [50]. Immune responses were detected at each dose level. Furthermore, 7 of 22 patients experienced an increase in PSA doubling time [50]. In a separate phase 1 trial, two different schedules of pTVG-HP were assessed in 17 patients [51]. The first schedule was fixed, with six 14-day interval immunizations, followed by boosters every 3 months for up to 2 years. The second schedule received 6 immunizations at 14-day intervals, after which they were monitored for PAP-specific immune responses to guide subsequent immunizations. The study identified that multiple DNA immunizations were required to elicit and maintain a long-term PAP-specific immune response. Given these findings, a randomized phase 2 study (NCT01341652) was conducted to determine whether vaccination could delay the development of metastatic disease in patients with biochemically recurrent disease [12]. Ninety-nine patients with castration-sensitive prostate cancer, without radiographic evidence of metastases and a PSA doubling time of less than 12 months, were randomized to receive pTVG-HP and GM-CSF adjuvant or GM-CSF alone. The results did not show an overall difference in 2-year metastasis-free survival between the two cohorts. However, a subset of patients with rapidly progressing disease (as determined by rapid PSA doubling time) and treated with pTVG-HP were identified to have longer metastasis-free survival (MFS) [12]. As an exploratory endpoint, sodium fluoride (^18^F-NaF) PET/CT was used to identify micrometastatic bone disease in a subset of patients. Decreases in NaF uptake were observed in pTVG-HP-treated individuals, compared with increases in the GM-CSF control group, suggesting that vaccination had detectable effects on micrometastatic bone disease. Overall, however, the conclusion of this study was that, while this vaccine demonstrated some antitumor efficacy in a subset of patients with rapidly progressing disease, it should not be further pursued as a single agent because the study did not meet the primary endpoint of significantly increased 2-year metastasis-free survival in patients treated with pTVG-HP [12]. Thus, it was deemed that DNA vaccines best be evaluated in combination with other vaccines or other immune-activating agents.

PSA is a classical biomarker for prostate cancer and is currently used as a measure of tumor response or progression. Given the increased expression of PSA in the tumor during disease progression, it has been explored as a possible target for DNA vaccines. In a phase 1 dose-escalation study, a plasmid encoding full-length PSA was given to patients with advanced CRPC in monthly cycles for 5 months. The doses given were 100, 300, and 900 μg of DNA, with 90% of the dose given intramuscularly and 10% of the dose given intradermally. Only the highest dose of 900 μg of DNA elicited PSA-specific cellular and humoral antibody responses [15]. No IL-10 was detected, however, other Th2 cytokines such as IL-4 and IL-6 were detected, suggesting that it is important to not only look at Th1 cytokines but also at potentially immunosuppressive cytokines [13]. To further improve immunogenicity of the PSA vaccine, researchers tested a DNA vaccine encoding the Rhesus PSA gene, which was administered with electroporation to increase transfection of the antigen-presenting cells [47]. Fifteen patients were required to start androgen deprivation therapy before vaccination. Patients received doses of plasmid ranging from 50 to 1600 μg. The DNA vaccine was administered intradermally, followed immediately by electroporation. All patients except one had pre-existing PSA-specific T cells, the frequency of which was increased by either androgen deprivation or by vaccination. However, only the two highest doses, 1000 and 1600 μg elicited immune responses [47].

Prostate-specific membrane antigen (PSMA) has also been tested as a target antigen for vaccination. PSMA is highly expressed in the epithelium in prostate adenocarcinoma [55], making it a promising target for vaccination. A DNA vaccine encoding an HLA-A2 binding epitope of the PSMA gene fused to a fragment of the tetanus toxin was tested in a phase 1/2 dose escalation trial [56]. Patients who were HLA-A2+ were recruited to receive vaccine with or without electroporation, whereas a patient population who were HLA-A2− served as a negative control. Vaccinated patients had a significant increase in PSA doubling time compared to unvaccinated patients, suggesting slower disease progression [56]. In patients that received the vaccine, PSMA-specific T cells were significantly increased postvaccination compared to baseline. A more recent study (NCT02514213) examined the effect of INO-5150, a DNA vaccine consisting of plasmids encoding both PSMA and PSA in patients with biochemically recurrent prostate cancer [48]. Treatment was considered safe with no patients experiencing serious treatment-related adverse events. A quarter of patients in the trial exhibited higher immune responses compared to baseline, to either PSMA or PSA, as detected by IFN-γ ELISPOT or by flow cytometric analysis of antigen-specific T cells.

The androgen receptor is expressed in the prostate and is important for prostate development, function and also plays a role in cancer progression, with AR overexpression identified as a major mechanism of resistance in CRPC [57]. A DNA vaccine encoding the ligand-binding domain of the androgen receptor (pTVG-AR) was demonstrated to delay tumor growth in several prostate cancer models [58]. In a phase 1 clinical trial (NCT02411786), pTVG-AR was given to patients with castration-sensitive prostate cancer, on two schedules and with or without the adjuvant GM-CSF. Vaccination was demonstrated to be safe, and one schedule was demonstrated to elicit more AR-specific, interferon-γ (IFN-γ) producing T cells compared to the other schedule [44]. The addition of GM-CSF did not significantly boost AR-specific immunity. Intriguingly, patients who developed cellular immune responses had significantly longer PSA progression-free survival compared to patients who did not develop immune responses. Further evaluation of this vaccine in combination with other agents is anticipated.

**Figure 1:**
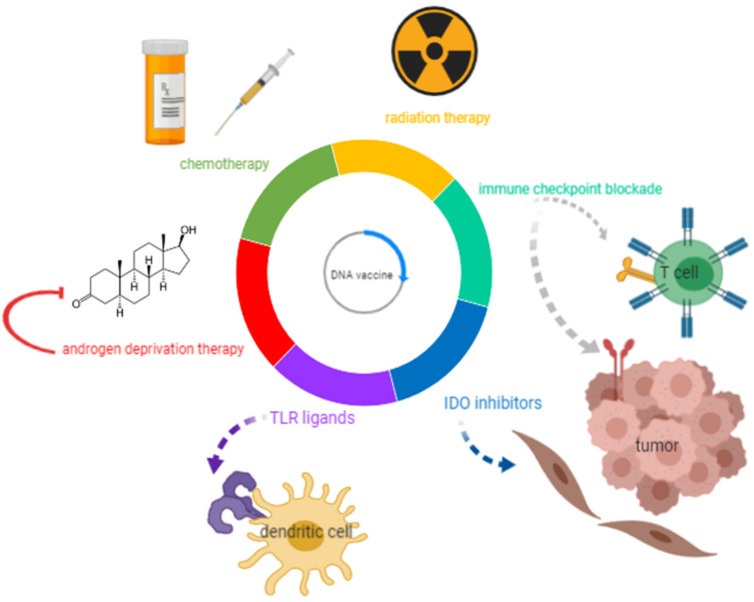
DNA vaccine combinations under evaluation as potential treatments for prostate cancer. With the goal of augmenting the efficacy of DNA vaccines in controlling prostate tumor growth, this review explores DNA vaccine combinations under investigation with current therapies for metastatic castration-resistant prostate cancer (mCRPC) (androgen deprivation, chemotherapy, and radiation), as well as immunomodulatory agents (immune checkpoint blockade, Toll-like receptors (TLR) ligands, and indoleamine 2,3 dioxygenase (IDO) inhibitors).

These recent and ongoing studies demonstrate that DNA vaccines are safe, can elicit antigen-specific T-cells, and can have other detectable antitumor effects. However, the direct antitumor effects as measured by changes in serum PSA or objective responses have been underwhelming when these agents have been used as monotherapies. Consequently, these approaches are currently being combined with other immunomodulatory therapies and conventional prostate cancer treatments to determine if there might be synergies that could be exploited to further improve the immunogenicity of DNA vaccines and augment the antitumor response. These approaches are depicted in Figure 1, and the mechanisms of action and evidence supporting their combination with DNA vaccines are summarized in the following sections.

## 3. Androgen Deprivation Therapy

Androgen deprivation therapy (ADT) has been a cornerstone therapy for the treatment of recurrent and metastatic prostate cancer. Early studies showed that androgen deprivation, either by surgical or chemical means, causes a decrease in prostate size and induces tumor apoptosis [59]. Androgen deprivation also affects the immune system in various ways. Castration causes an increase in thymus size, whereas testosterone replacement after castration induces thymic regression [60]. Spleens from castrated C57Bl/6 mice have significantly more B cells and significantly fewer CD4+ T cells [61]. Androgen deprivation also causes increased T-cell infiltration into the prostate and prostate tumors. In rodent studies, these infiltrating lymphocytes were initially of Th1 phenotype within the first 30 days after castration, but by 90 days after castration, the infiltrating lymphocytes had a Th17 phenotype [62,63,64]. In human studies, tumor-infiltrating lymphocytes consist of both effector CD8+ T cells and CD4+ T cells, including regulatory T cells (Treg) [65].

With these different immunomodulatory effects on the immune system, ADT may be logically combined with vaccine-based therapies. In a phase 2 clinical trial assessing the sequencing of Sip-T and androgen deprivation in patients with biochemically recurrent prostate cancer at high risk of metastasis, patients who received Sip-T followed by ADT had increased IFN-γ responses to PA2024 (the PAP/GM-CSF fusion protein vaccine antigen) compared to patients treated with ADT followed by Sip-T at the 6 week time point and increased PA2024-specific T cell proliferation averaged over all time points [66]. In another randomized trial comparing viral PROSTVAC vaccine to the AR antagonist nilutamide, in patients with biochemically recurrent castration resistant prostate cancer, there was no significant difference in time to treatment failure between the vaccine arm versus the nilutamide arm [67]. However, upon PSA progression, patients were offered the combination therapy, adding either PROSTVAC or nilutamide to their treatment regimen. Patients who received PROSTVAC followed by nilutamide had a significantly longer median time to treatment failure of 13.9 months, compared to patients who received nilutamide followed by PROSTVAC with a median time to treatment failure of 5.2 months [67]. The same patients were followed-up 6 years later, and the trend held. Patients that received PROSTVAC first followed by nilutamide had a median overall survival of 6.2 years, significantly longer than patients who received nilutamide first followed by PROSTVAC who had a median overall survival of 3.7 years [68]. A recent study examining GVAX with the T-reg depleting agent cyclophosphamide followed by degarelix, showed that the triple combination resulted in significantly increased time to PSA progression and time to next treatment compared to degarelix alone [69]. Treatment with ADT resulted in CD8+ T cell infiltration into the tumor, however, also increased T-regs and likely immunosuppressive myeloid cells [69].

Androgen deprivation therapy specifically in combination with DNA vaccines has been less explored. Preclinical studies in mouse and rat models using the pTVG-AR DNA vaccine described earlier demonstrated significant antitumor activity when androgen deprivation was combined with vaccination, likely due to increased expression of the androgen receptor within prostate tumors following androgen deprivation [57]. As discussed, this DNA vaccine was evaluated in a phase 1 clinical trial in patients who had recently started ADT. Patients who developed immunity to AR were found to have a prolonged time to castration resistance, consistent with prior rodent studies [44]. Collectively, these data suggest that DNA vaccines should be further explored in combination with androgen deprivation and that the type of androgen deprivation and sequence with vaccination should be further studied.

## 4. Chemotherapy

Chemotherapy is an overarching term for any cytotoxic drug that targets rapidly dividing cells, including cancer cells. For many years, chemotherapy was the only option for patients with progressive mCRPC. Docetaxel is still considered a first line of treatment for mCRPC, offering a 2–3 months median survival advantage compared to mitoxantrone [70]. A second-generation cytotoxic chemotherapy, cabazitaxel, demonstrated a 2.4-months increase in median survival for patients previously treated with docetaxel, and was approved for patients with mCRPC [71]. Although the survival advantage with chemotherapy is limited for patients with castration-resistant disease, it has been shown to be effective in pain management and improving patient quality of life.

Investigators have long thought that chemotherapy and anticancer vaccines were incompatible due to the immunosuppressive effects of chemotherapy drugs. However, mounting evidence suggests this might not be the case [72]. A preclinical study examining the effects of docetaxel and a poxviral vaccine encoding the self-antigen carcinoembryonic antigen (CEA) in tumor-bearing mice demonstrated that when docetaxel was administered after the vaccine, there was an increased antitumor effect compared to vaccine alone [73]. In a phase 2 clinical trial that assessed whether docetaxel concurrently administered with PROSTVAC could elicit an immune response, there was no observed decrease in T cell responses to PSA in the docetaxel/vaccine group compared to vaccine alone, suggesting chemotherapy did not impair immune responses to vaccine [74]. A randomized multicenter phase 2 trial to evaluate PROSTVAC prior to docetaxel chemotherapy, versus docetaxel chemotherapy alone, was planned to evaluate whether vaccine could improve the overall survival from chemotherapy alone. Unfortunately, this trial was stopped early without meeting its accrual goal [75]. In another small phase 2 clinical trial, high-risk patients with localized disease were treated with neoadjuvant docetaxel and GVAX, followed by radical prostatectomy [76]. Six patients completed the treatment regimen. No serious drug-related adverse events were observed and the median change in prostate-specific antigen (PSA) was minor but decreasing. Of the five patients who completed prostatectomy, four had a down-staging of their Gleason score. Undetectable PSA was achieved in three patients at 2 months after prostatectomy and in two patients at 3 years after prostatectomy, demonstrating that chemoimmuno therapy combinations can result in durable responses possibly due to the disruption of the immunosuppressive microenvironment. Two phase 3 trials examining the combination of docetaxel with GVAX were terminated prematurely due to increased death in the combination arm [77]. Such findings reinforce the need for basic scientific research into the mechanisms of potential interactions and importance of sequence when combining therapies. To our knowledge, the specific combination of chemotherapy with DNA vaccines has not yet been explored in prostate cancer or indeed, in any type of cancer.

## 5. Radiation Therapy

Radiation therapy has long been a standard treatment for localized prostate cancer by means of external beam radiation therapy (EBRT) or brachytherapy. Several bone-targeting radioactive metals, including strontium-89 and samarium-153, have been used in the treatment of bone-metastatic prostate cancer. In 2013, the alpha particle emitting compound radium-223 (Xofigo^®^) was approved based on increased overall survival compared to placebo in patients with bone-metastatic CRPC [78]. This agent accumulates in areas of active bone turnover, effectively targeting radiation to bone metastatic sites [79]. More recently, a lutetium-177-labeled small molecule targeting prostate membrane specific antigen (PSMA-617) has been developed, with the goal of delivering radiation therapy more specifically to all sites of metastatic disease. Early clinical trials have demonstrated activity of this agent [80].

Many groups have demonstrated that radiation therapy has immunostimulatory effects including increased MHC I expression within tumors [81,82], increased cross-presentation by antigen-presenting cells [83], immunogenic cell death [84], and inflammatory cytokine production [85]. In addition, studies suggest that much of the antitumor efficacy of radiation is actually mediated by CD8+ T cells, as radiation therapy is less effective in murine models in the absence of functional CD8+ T cells [86]. There are a wide variety of preclinical data showing that radiation can sensitize tumors to various forms of immunotherapy including vaccines [87,88,89,90].

A handful of preclinical studies have examined the combination of radiation with DNA vaccination, albeit in non-prostate cancer models. For example, the combination of a DNA vaccine encoding Mucin 1, pcDNA3-hMUC1, with systemic ^131^I was able to significantly improve survival in mice bearing CT26 colon tumors [91]. Uptake of the radioiodine was dependent on transducing the colon cancer line to express the sodium iodide symporter, potentially limiting the broader application of this strategy [91]. Another group demonstrated that the combination of local radiation therapy with DNA vaccination encoding CTGF/E7 can lead to systemic immune responses in the TC-1 murine model of HPV-expressing tumors [92]. Delivering moderate doses (6 Gy) of EBRT to the primary tumor together with the DNA vaccine resulted in increased antitumor efficacy both at the primary tumor and at a distant secondary tumor. Furthermore, both sites had increased CD8+ T cell infiltration and increased numbers of mature dendritic cells [92]. These studies demonstrate the potential of this therapeutic combination in improving antitumor responses. However, the combination of local or systemic radiotherapy with DNA vaccines in prostate cancer is currently underexplored.

## 6. Immune Checkpoint Blockade

One of the most exciting areas in cancer immunotherapy has been immune checkpoint blockade, with the 2018 Nobel Prize in Medicine awarded to James Allison and Tasuku Honjo for their roles in the discovery of two targets for immune checkpoint therapy, CTLA-4 and PD-1 [93]. CTLA-4 (or CD152) is expressed on T cells and competes with CD80/86 to prevent the costimulation of CD8+ T cells and inhibit T cell activation [94]. Anti-CTLA-4 therapy acts by allowing this costimulation to occur and has also been reported to deplete intratumoral Tregs that express CTLA-4 [95,96]. PD-1 is expressed on activated T cells, and PD-1 signaling can be activated by its ligands, PD-L1 or PD-L2, expressed on tumor and immune cells [97,98], which causes downregulation of T cell effector function [99]. Immune checkpoint blockade monotherapy has shown remarkable clinical efficacy in a variety of cancer types including melanoma [100], renal cell carcinoma [101], and nonsmall cell lung cancer [102]. Currently, many other immune checkpoints are under investigation, which will likely lead to the development of many other immune checkpoint inhibitors for eventual clinical use.

In patients with mCRPC, multiple studies of either PD-1 or CTLA-4 blockade as monotherapies have failed to demonstrate significant numbers of objective response or improvements in overall survival [3,4]. Even in cancers with evidence of efficacy, typically a minority of patients respond to monotherapy. One approach to improve the response rate has been to use blockade of multiple checkpoints simultaneously. Clinical trials in other cancers have shown synergy when both PD-1 and CTLA-4 are blocked simultaneously, however, with concomitant increases in toxicity [103,104,105,106]. To date, early trials with anti-PD-1 and anti-CTLA-4 combination checkpoint blockade have demonstrated little benefit in patients with mCRPC, except perhaps in patients with DNA damage repair mutations [107].

Because T-cell checkpoint blockade therapies rely on the presence of tumor-specific T cells, the paucity of prostate tumor-specific mutations and tumor-infiltrating lymphocytes has been attributed to the lack of response of prostate cancer to these treatments. Conceptually, therefore, the addition of vaccination strategies may overcome resistance to checkpoint blockade by activating or expanding tumor-associated T-cell populations on which these agents act. There have been many preclinical studies that support the potential for combining vaccination and immune checkpoint blockade in a variety of models. As described above, the GVAX cellular vaccine was evaluated alone and in combination with anti-CTLA-4 blocking antibodies in the TRAMP murine model of prostate cancer, and it demonstrated antitumor activity only with the combination treatment [22]. Similar studies have been conducted by others, demonstrating that this combination can lead to increased tumor-infiltrating CD8+ lymphocytes [108]. As described earlier, this approach was also evaluated in a small clinical trial for patients with mCRPC and similarly suggested benefit with this combination [23].

Similar findings have been found using DNA vaccines in multiple preclinical models. For example, in the P815 murine mastocytoma model expressing the tumor-associated antigen P1A, and treated with a DNA vaccine encoding P1A, tumor growth was significantly suppressed compared to the control [109]. When the DNA vaccine was combined with CTLA-4 and PD-1 blockade, they saw significantly improved survival over the DNA vaccine alone [109]. In another study, the same group used the B16F10-OVA tumor model and treated them with DNA vaccines encoding OVA (pOVA) or IL-12 (pIL-12), with or without CTLA-4 or PD-1 blockade. The group treated with pOVA + pIL-12 + dual checkpoint blockade had the most delayed tumor growth, compared to any agent alone [110]. Another group used a DNA vaccine against telomerase reverse transcriptase (TERT) in mice bearing TC-1 tumors. When the TERT DNA vaccine was combined with either PD-1 or CTLA-4 blockade, tumor growth was significantly slowed compared to the control [111]. Finally, a DNA vaccine encoding the prostate tumor antigen SSX-2 was found to increase the expression of PD-1 on vaccine-activated CD8+ T cells following vaccination, and the efficacy of this vaccine could be increased in tumor-bearing mice when combined with PD-1 or PD-L1 blockade [112].

In addition to extensive preclinical literature exploring these combinations, there are several ongoing and completed clinical trials examining DNA vaccination in combination with immune checkpoint blockade in prostate cancer patients. In a pilot clinical trial conducted by our group examining pTVG-HP combined with pembrolizumab, this 26-patient, two-arm study examined whether PD-1 blockade was more effective when used at the time of vaccination with pTVG-HP or after the vaccination schedule had ended [42]. We observed increased PAP-specific IFN-γ and granzyme B responses in both arms in comparison to baseline values, however, patients who received concurrent PD-1 blockade with vaccination had greater responses in serum PSA, tumor volume reduction, and CD8+ T cell-infiltration in comparison to the patients who received pembrolizumab after the vaccination schedule ended [42]. Because clinical responses were only detected in patients who developed antigen-specific immunity, a separate phase 2 study (NCT04090528) is currently underway evaluating whether immunization with two DNA vaccines along with pembrolizumab is better than vaccination against a single antigenic target. The vaccines being tested in this trial are pTVG-HP and pTVG-AR. Another ongoing phase 2 study (NCT03600350) is evaluating a similar approach in patients with nonmetastatic, PSA-recurrent prostate cancer. This study aims to investigate the effects of pTVG-HP in combination with nivolumab (anti-PD-1 antibody) to determine whether the treatment can eradicate disease in patients with early biochemically recurrent disease. Finally, a pilot phase 1 study (NCT03532217) is recruiting patients with metastatic castration-sensitive prostate cancer to investigate the effects of PROSTVAC in combination with a DNA vaccine encoding patient-specific neoantigens and checkpoint blockade (anti-CTLA-4 plus anti-PD-1). The primary objectives of this study include safety, tolerability, and immune responses. With these exciting advances, it is hoped that the combination of DNA vaccines with immune checkpoint blockade will lead to more effective treatment and management of CRPC.

## 7. Toll-Like Receptor (TLR) Ligands

TLRs are transmembrane receptor proteins involved in innate immunity. Recognition of specific molecules with pathogen-associated molecular patterns (PAMPs) or danger-associated molecular patterns (DAMPs) triggers changes in many types of immune cells [113]. In humans, 10 different TLRs have been identified (TLR1-10), and they are expressed on macrophages and dendritic cells, as well as nonimmune cells, such as epithelial cells or fibroblasts [114]. Each TLR recognizes different biomolecules, and stimulation of these receptors leads to the activation of innate and adaptive immune responses. TLR stimulation also affects the activation of T cells. TLR ligands can suppress activation-induced PD-1 expression on CD8+ T cells via acting on professional antigen-presenting cells to secrete IL-12 [115,116], as well as directly affect T-cell activation by regulating T-cell energy metabolism through favorable changes in glycolysis and glutaminolysis [117]. Together, these suggest potential synergy by combining TLR ligands as adjuvants with DNA vaccines.

Adjuvants can boost antigen-specific immune responses, and thus ligands for TLR have been investigated as adjuvants for traditional vaccines [118]. Various in vivo studies have demonstrated the potential of TLR ligands as tumor vaccine adjuvants. For example, treatment of HPV-driven tumors with HPV E7 oncoprotein-derived peptide vaccines combined with TLR3 or TLR9 ligands showed T-cell activation and subsequent tumor regression in vivo [119]. Similarly, melanoma-specific peptide vaccines with TLR9 agonists also demonstrated tumor antigen-specific T-cell activation and improved antitumor response in mouse models [120]. The efficacy of TLR stimulation when delivered with DNA vaccines has been also investigated. T-cell activation by a DNA vaccine encoding HPV E7 and combined with TLR4 ligands augmented antitumor responses in a mouse tumor model [121], and the combination of DNA vaccine with TLR3 or 7 ligands also showed increased antitumor responses [122].

To our knowledge, only two clinical trials are currently investigating the combination of DNA vaccines and TLR ligands. One phase 1 clinical trial is examining the safety of imiquimod, a TLR7 ligand, and a DNA vaccine (VCX-3100, encoding genes E6 and E7 from the human papillomavirus types 16 and 18) in patients with cervical intraepithelial neoplasia level 3 (CIN3) (NCT03206138). Primary data has yet to be reported, however, the primary endpoint is to determine the ratio of patients who have regression of cervical lesions to CIN1 or less. A separate, ongoing phase 2 clinical trial is assessing the vaccine VCX-3100 combined with imiquimod in patients with vulvar intraepithelial neoplasia associated with HPV16/18 (NCT03180684). Although studies into the combinations of DNA vaccines with TLR ligands are still in their infancy, they could potentially be another way to augment immunogenicity and hence efficacy of DNA vaccines.

## 8. IDO Inhibitors

Indoleamine 2,3 dioxygenase (IDO), an IFN-γ-inducible tryptophan metabolizing enzyme, is one of the many factors produced by tumors and other immune cells that have known immunosuppressive properties [123,124]. IDO is a catabolic enzyme responsible for the degradation of tryptophan, and the degradation products such as kynurenine, which is known to have substantial inhibitory effects in the microenvironment of solid tumors [125]. IDO may inhibit intratumoral T-cell activity through the depletion of tryptophan in the tumor microenvironment or by recruiting immunosuppressive cells such as MDSCs and Tregs through activation of the aryl hydrocarbon receptor (AhR) by kynurenine [126]. In addition, the expression of IDO has been established as one potential mechanism of resistance to CTLA-4 blockade therapy, spurring the clinical development of IDO inhibitors as potential anticancer therapies to combine with T-cell checkpoint inhibitors [127]. IDO inhibitors such as epacadostat have been explored with the CTLA-4 checkpoint inhibitor ipilimumab in clinical trials for the treatment of melanoma [128]. In a phase 1 clinical trial, the combination of epacadostat with ipilimumab had an acceptable toxicity profile, as well as potentially enhancing the action of ipilimumab in patients with metastatic melanoma [128]. A large phase 3 trial evaluating pembrolizumab with or without epacadostat, however, failed to demonstrate a benefit in patients with advanced melanoma [129].

IDO expression was detected in murine TRAMP prostate tumors and was associated with early prostate cancer progression, suggesting it might be a therapeutic target for prostate cancer [130]. IDO gene expression has been detected in human prostate cancers [131], however, IDO activity is more typically measured by serum kynurenine and tryptophan levels, yielding a (kyn:trp) ratio. This ratio has been debated as a biomarker of prostate cancer detection or progression [132,133]. In recent studies, we assessed serum kyn:trp ratios from patients with different stages of prostate cancer and found that IDO activity increased with stage of disease [10]. This activity, and IDO expression in tumors, was markedly increased following treatment with an antitumor DNA vaccine and/or PD-1 blockade. Furthermore, IDO activity was found to suppress the function of vaccine-induced T cells and to be highest in patients who did not demonstrate benefit from immunotherapy, suggesting that IDO blockade might best be used in combination with vaccination [10].

To date, very few studies have examined the combination of DNA vaccination with IDO inhibition. One study examined tumor growth after DNA vaccination and IDO silencing by siRNA [134]. Mice were implanted with MBT-2 tumor, which overexpresses Her2/neu [135]. They were then treated with either a DNA vaccine encoding neu, siRNA for IDO, or a fusion plasmid that encodes both neu and IDO siRNA. When tumor-bearing mice were immunized with the neu DNA plasmid or IDO siRNA alone, tumor growth was significantly slower compared to the control, but when mice were treated with the fusion neu-IDO siRNA plasmid, the mice had significantly longer survival compared to either neu vaccine or IDO siRNA alone [134]. Further studies evaluating the combination of IDO inhibitors with antitumor DNA vaccines are anticipated.

## 9. Future Directions

The future of DNA vaccines in combination with other therapies for the treatment of prostate cancer is promising, however, many factors require further investigation. For example, resistance to immunotherapy is likely due to the presence of an immunosuppressive tumor microenvironment. Factors that can contribute to immunosuppression include cell populations such as Tregs, MDSCs, and likely other cell types that have yet to be identified. Recently, a cell population identified as CD4+ Foxp3-PD-1^hi^ was demonstrated to suppress tumor-infiltrating and peripheral CD4+ and CD8+ effector T cells [136]. These cells, which are molecularly similar to follicular helper T cells (T_FH_), were shown to be induced by CTLA-4 blockade and repressed by PD-1 blockade [136]. Hence further evaluation of the tumor microenvironment, with characterization of the location, type, and phenotype of infiltrating immune cells and immunosuppressive mechanisms, will most likely help predict which immunotherapies (or combinations) will be most effective.

With the multiple agents approved for the treatment of prostate cancer, and the various potential combinations described above, the scheduling of combination treatments must also be addressed. This is particularly important, as preclinical and clinical evidence has suggested that antitumor vaccines may be most effective when used prior to androgen deprivation [66,67,68,69], chemotherapy [73,74,76], or immune checkpoint blockade [110,137]. In addition, the effects of other therapies on effector or regulatory components of the immune system need to be more comprehensively addressed.

Finally, there have been extensive efforts to predict which patients will respond to immunotherapies mainly focusing on immune checkpoint inhibitors [138]. Biomarkers evaluating the tumor microenvironment, such as tumor PD-L1 expression, T-cell infiltration, cytokine signatures, and IDO expression have correlated with positive responses to immune checkpoint blockade [138]. Even though immune checkpoint inhibitors as a monotherapy have shown modest responses in prostate cancer patients, these biomarkers, and early changes in these biomarkers, should be considered and may provide valuable insight to patients who may benefit with combination therapies [4,139,140].

## 10. Conclusions and Future Perspective

DNA vaccines have great potential to improve cancer treatment in patients with metastatic castration-resistant prostate cancer. They can be used to target common cancer antigens, making them ideal for off-the-shelf applications, as compared to personalized treatments such as Sip-T. DNA vaccines can also be easily modified to encode unique mutation-associated antigen targets, using conventional cloning techniques. DNA vaccines offer many manufacturing advantages over other vaccine approaches in terms of cost, scalability, and stability. However, despite all these advantages, DNA vaccines used as monotherapies, like other vaccine approaches, have demonstrated modest outcomes in clinical trials, suggesting their best use will be in combination with other agents. To date, combination with both conventional and immunomodulatory therapies show great promise for the treatment of mCRPC. However, further studies must be done to investigate the immunological resistance mechanisms within the tumor microenvironment, identify how approved and investigational agents can be rationally combined and scheduled to improve antitumor efficacy, and evaluate markers that can help identify the individuals that will most likely respond to these treatments.

## Figures and Tables

**Table 1:** Clinical trials with DNA vaccines (both as monotherapy and in combination) in prostate cancer to date.

Vaccine Antigen	Vaccine Name	Combination	Phase (Number Treated)	Rationale/Approach	Major Finding	*NCT Trial Number*	Ref.
Rhesus PSA	pVAXrcPSAv531	–	1 (*N* = 15)	Dose escalation. Safety, changes in PSA kinetics, and detection of PSA-specific immune responses in patients with nmCSPC	Vx was safe. No changes in PSA kinetics. 14/15 patients had PSA-specific immune responses due to vx or ADT	*NCT00859729*	[47]
PSA	pVAX/PSA	–	1 (*N* = 8)	Dose escalation. Safety and detection of PSA-specific cellular immunity in CRPC	Vx was safe. At highest dose (900 μg), PSA-specific cellular and humoral immunity detected	*-*	[15]
PSA + PSMA	INO-5150	+ IL-12 DNA plasmid (INO-9012)	1 (*N* = 62)	Safety, tolerability, immune response to PSA and PSMA, PSA doubling time and PSA kinetics. Patients with biochemically recurrent PCa (nmCSPC)	Vx was safe, and 53/62 patients were progression-free after 72 weeks. PSA doubling time increased in patients with pretreatment PSA doubling time <12 months, and 47/62 patients had PSA- or PSMA-specific immunity	*NCT02514213*	[48]
PSMA + PRAME	MKC1106-PP	–	1 (*N* = 24)	Fixed DNA plasmid (prime) and two different doses of peptide boost (low/high). Safety, PSA or PRAME specific immune response, clinical benefit (stable disease) in CRPC	Vx was safe, and 4/10 showed PSA decline or stable disease for >6 months. Association between antigen-specific T cells above baseline and disease control (stable disease >6 months)	*NCT00423254*	[45]
NY-ESO1	pPJV7611	–	1 (*N* = 16)	Safety and immune response in patients with different malignancies, including 9 with metastatic prostate cancer	Vx was safe. All 10 patients had CD4+ immune responses, and 2/10 patients had CD8+ immune responses	*NCT00199849*	[49]
AR LBD	pTVG-AR (MVI-118)	–	1 (*N* = 40)	Safety, immune response, median time to PSA progression, and 18-month PSA progression free survival in patients with mCSPC	Vx was safe, and 14/30 evaluated patients developed AR-specific cellular immunity. Patients with T cell immunity had significantly longer time to PSA progression	*NCT02411786*	[44]
PAP	pTVG-HP (MVI-816)	–	1 (*N* = 22)	Dose escalation. Safety, PAP-specific immune response, PSA doubling time in patients with nmCSPC	Vx was safe, and 9/22 patients developed PAP-specific CD4+ and/or CD8+ cell proliferation. PSA doubling time increased from 6.5 months pretreatment to 8.5 months post-treatment and 9.3 months to 1-year post-treatment	*NCT00582140*	[50]
PAP	pTVG-HP (MVI-816)	–	1/2 (*N* = 16)	Tested two schedules: 6 immunizations every 2 weeks, then every 3 months for up to 2 years versus 6 immunizations every 2 weeks, then immunized based on results from immune monitoring. In patients with nmCRPC	Immune monitoring did not lead to superior schedule. Antigen-specific T cells elicited persisted over time	*NCT00849121*	[51]
PAP	pTVG-HP (MVI-816)	–	2 (*N* = 99)	Randomized to pTVG-HP with GM-CSF versus GM-CSF alone in patients with nmCSPC and PSA doubling time < 12 months	Two-year metastasis-free survival was not different overall between study arms. Patients with a pretreatment PSA doubling time < 3 months, MFS was significantly longer in vx arm. Decreased NaF uptake by PET/CT imaging suggested vx affected bone micrometastatic disease	*NCT01341652*	[12]
PAP	pTVG-HP (MVI-816)	+ pembrolizumab	1/2 (*N* = 66)	Assess pTVG-HP with pembrolizumab (concurrent) or pTVG-HP vx first followed by pembrolizumab (sequential) in patients with mCRPC	Median time to radiographic progression was not different; 8/13 patients treated concurrently and 1/12 patients treated sequentially had PSA declines from baseline. PSA declines associated with PAP-specific cellular immunity and CD8+ tumor infiltration. Expansion cohorts ongoing	*NCT02499835*	[42,52]
PAP	pTVG-HP (MVI-816)	+ Sip-T	2 (*N* = 18)	Assessed whether pTVG-HP could augment Sip-T antitumor efficacy in patients with mCRPC	Treatment was safe, and 11/18 patients developed PAP-specific cellular immunity. Higher antibody immunity observed in patients receiving pTVG-HP boost compared to Sip-T alone. Median time to progression was not significantly different	*NCT01706458*	[53]
PAP	pTVG-HP (MVI-816)	+ nivolumab	2 (*N* = 21–41)	Assess the safety and PSA complete response rate using pTVG-HP with nivolumab in patients with nmCSPC	Ongoing	*NCT03600350*	–
PAP and AR LBD	pTVG-HP (MVI-816) and pTVG-AR (MVI-118)	+ pembrolizumab	2 (*N* = 60)	Assess efficacy (6m PFS) of one versus two DNA vaccines, with PD-1 blockade in patients with mCRPC	Ongoing	*NCT04090528*	–
Mutation-associated neoantigens		+ PROSTVAC + ipilimumab + nivolumab	1 (*N* = 20)	Will elucidate safety and immune response to a shared antigen vaccine and tumor-specific antigen DNA vaccine with ICB	Ongoing	*NCT03532217*	–

**Abbreviations used:** ICB immune checkpoint blockade, mCRPC metastatic castration-resistant prostate cancer, nmCSPC non-metastatic castration-sensitive prostate cancer, MFS metastasis-free survival, NaF sodium fluoride, PCa prostate cancer, vx vaccination.

## Abbreviations

ADT	androgen deprivation therapy
AR	androgen receptor
EBRT	external beam radiation therapy
GM-CSF	granulocyte-macrophage colony stimulating factor
GVAX	cancer vaccine consisting of whole tumor cells that secrete GM-CSF
IDO	indoleamine 2,3 dioxygenase
mCRPC	metastatic castration-resistant prostate cancer
MDSC	myeloid-derived suppressor cells
MFS	metastasis-free survival
NaF	sodium fluoride
PAP	prostatic acid phosphatase
PROSTVAC	prostate cancer vaccine consisting of vaccinia encoding PSA as a primary immunization followed by fowlpox encoding PSA booster immunizations
PSA	prostate-specific antigen
PSMA	prostate-specific membrane antigen
Sip-T	sipuleucel-T, an FDA-approved autologous cellular vaccine for the treatment of prostate cancer
TRAMP	transgenic adenocarcinoma of the mouse prostate mouse model
Treg	regulatory T cells
